# Promoting Secondary Analysis of Electronic Medical Records in China: Summary of the PLAGH-MIT Critical Data Conference and Health Datathon

**DOI:** 10.2196/medinform.7380

**Published:** 2017-11-14

**Authors:** Peiyao Li, Chen Xie, Tom Pollard, Alistair Edward William Johnson, Desen Cao, Hongjun Kang, Hong Liang, Yuezhou Zhang, Xiaoli Liu, Yong Fan, Yuan Zhang, Wanguo Xue, Lixin Xie, Leo Anthony Celi, Zhengbo Zhang

**Affiliations:** ^1^ Department of Biomedical Engineering Chinese People’s Liberation Army General Hospital Beijing China; ^2^ Laboratory of Computational Physiology Institute for Medical Engineering and Science Massachusetts Institute of Technology Cambridge, MA United States; ^3^ Department of Critical Care Medicine Chinese People’s Liberation Army General Hospital Beijing China; ^4^ School of Biological Science and Medical Engineering Beihang University Beijing China; ^5^ Chinese People’s Liberation Army Medical School Beijing China; ^6^ Department of Computer Application and Management Chinese People’s Liberation Army Medical School Beijing China; ^7^ Department of Respiratory and Critical Care Medicine Chinese People’s Liberation Army General Hospital Beijing China

**Keywords:** electronic health record, datathon, database, intensive care units

## Abstract

Electronic health records (EHRs) have been widely adopted among modern hospitals to collect and track clinical data. Secondary analysis of EHRs could complement the traditional randomized control trial (RCT) research model. However, most researchers in China lack either the technical expertise or the resources needed to utilize EHRs as a resource. In addition, a climate of cross-disciplinary collaboration to gain insights from EHRs, a crucial component of a learning healthcare system, is not prevalent. To address these issues, members from the Massachusetts Institute of Technology (MIT) and the People’s Liberation Army General Hospital (PLAGH) organized the first clinical data conference and health datathon in China, which provided a platform for clinicians, statisticians, and data scientists to team up and address information gaps in the intensive care unit (ICU).

## The Potential of Healthcare Data

On June 21, 2016, the Chinese State Council promulgated its Guiding Opinions on Promoting and Regulating the Development of the Application of Healthcare Big Data. The report identified healthcare data to be a strategic national resource and that its development would significantly impact healthcare and medical treatment. It also raised the importance of gathering and utilizing healthcare data to a national level.

Healthcare data comes from many sources, giving rise to various fields (ie, medical and health informatics, translational bioinformatics, sensor informatics, and imaging informatics), and within each field, new analytic tools to understand health and disease [1]. In particular, the analysis of data contained within electronic health records (EHRs) is a promising avenue of research for clinicians and data scientists. EHRs contain large volumes of data regarding patient care, both structured and unstructured, making them highly valuable resources for knowledge discovery [2].

For decades, clinical research has relied on randomized controlled trials (RCTs) as the authoritative methodology—conclusions from RCTs are deemed inherently more reliable compared to those of observational studies. While medical societies rely on RCTs to develop clinical practice guidelines, they have some well-known and notable drawbacks [3]. They are costly, labor-intensive, generally take a long time to complete, and tend to contain restrictive inclusion and exclusion criteria, which leads to limited generalizability.

With the spread of EHRs across hospitals, there is an interest in harnessing the data contained therein to power longitudinal, population-based studies without the artificial conditions imposed by RCTs [4]. Once the data infrastructure is created, further research costs tend to be low and these databases lend themselves to iterative analyses—findings can be tested against a wide variety of populations, circumstances, potential confounders, and timeframes, all contained within the existing data. New insights pertinent to the day-to-day practice of clinicians may be gleaned from the troves of digital documentation [2,5]. Like RCTs, retrospective clinical research using EHRs should be hypothesis-driven. However, unlike RCTs, the variables that are included in the analysis are not limited to those pre-defined during the design phase of the RCT. In addition, post-hoc analysis of patient subsets in an RCT for hypothesis generation is limited by the fixed sample size. This is typically not the case for secondary analysis of EHRs. However, the absence of randomization makes retrospective studies problematic and requires more complex causal inference methodologies.

Compared to other countries, China enjoys several advantages when it comes to EHR-based clinical research. First, China has a large population, growing by 5.84% between 2000 and 2010 to 1.34 billion. To meet the needs of this growing population, China has a very large healthcare system [6]. The National Health and Family Planning Commission of the People’s Republic of China (NHFPC) statistics from June 2016 show that there were 989,403 health institutions in China, including 28,261 hospitals, 927,147 community health centers, and 30,814 specialized public health institutions [7,8]. In the first half of 2016, over 3.84 billion visits were made to these health institutions, including over 1.56 billion hospital visits [9]. The volume of health information generated by this enormous amount of healthcare consumption presents a great opportunity to understand determinants of patient outcomes and identify treatments most effective among the Chinese population.

China also has relatively robust clinical information systems. These systems have been built and refined since the 1997 launch of the Military No.1 project, which was invented and promoted by the People’s Liberation Army General Hospital (PLAGH). This project established a software framework with a set of standards targeted towards medium and large hospitals for hospital, staff, and patient administration, and was adopted by several hundred hospitals over the next decade. The primary functions of its EHR system included keeping track of patient visits, medical history, treatments, and drug prescriptions [10].

In the central government’s 12th Five-Year Plan (2011-2016), a series of social and economic development initiatives, the NHFPC declared its “3521” Project for electronic health (e-Health), which was subsequently revised to “4631-2.” Here, the “4” denotes the establishment of 4 levels of healthcare administration platforms: national, provincial, prefecture, and county. The “6” represents the strengthening of 6 key services: public health, medical services, medical security, drug administration, family planning, and integrated health management. The “3” denotes the development of 3 fundamental database systems: the electronic medical record, the EHR, and a national database containing the healthcare-related information of the entire population. The “1” denotes the establishment of one centralized communication network to integrate all the previously listed elements. The final “2” denotes the establishment of health information system standards and the protection of healthcare information [7,11,12]. With this government mandate, the EHR adoption rate increased from 25% in 2008 to 47% in 2013 [11].

## Challenges to Secondary Use of Health Data in China

Current EHRs are designed to support administrative and billing functions, frequently resulting in their inability to capture clinical information in a structured manner, not to mention poor usability, which hampers clinician efficiency.

The 2015 hospital information systems survey by the China Hospital Information Management Association (CHIMA) received comprehensive responses from 536 hospitals, which is about 2% of the national total [13]. In accordance with the NHFPC’s 3-tier classification system, there were 342 (63.8%, 342/536) tier 3 hospitals, and 194 (36.2%, 194/536) lower tier hospitals, where a higher tier indicates a more advanced hospital. The metrics used to define the tiers are hospital capacity based on staff and bed numbers, the level of technology employed, the equipment and facilities available, the quality of the management and logistics, and the quality of care. The tier distribution of the survey sample differs heavily from the national distribution: 12.4% tier 3 and 87.6% tier 2 or below. Hence, the patterns drawn from the survey give insight into China’s more technologically and financially advanced hospitals, rather than the national trend.

Among the surveyed hospitals, the most frequently used hospital information system services and the percentage of hospitals using each service are as follows: pharmaceutical administration (78%), emergency department billing and administration (77%), inpatient medication administration (77%), and outpatient scheduling (76%). Correspondingly, the most prevalent issues raised against the hospital information systems and the percentage of hospitals raising such issues were: insufficient interoperability and standards (53%), lack of flexibility and features to capture individual patient characteristics (48%), and a lack of human usability in the software layout (44%). Given this information, it is not surprising that a recent study of tertiary hospitals showed high EHR adoption rates (73%) but extremely low (1%) levels of data integration for informing clinical decision support [14].

The development of healthcare databases able to support secondary analysis is impeded by 2 skill shortages. The supply side issue is the lack of skilled information technology (IT) professionals needed to integrate EHR data to generate a research resource and that understand healthcare data. Just as important, is the lack of demand for such resources by potential clinical researchers in China due to a lack of familiarity with the field. According to an informal survey conducted with 37 staff members from the 20 departments of the PLAGH ahead of the datathon, clinicians admitted lacking knowledge and background about how to incorporate insights from digital health data into their clinical practice. Clinicians typically conduct clinical research independently or as the director of a group that includes a statistician. However, secondary analysis of EHRs requires a multidisciplinary team approach, with members appreciating each other’s contribution and acknowledging the limitations of their expertise [15]. Clinicians must be willing to embrace the uncertainties and information gaps in the practice of medicine and their limited understanding of machine learning, while data scientists must defer to clinicians in formulating relevant projects that can lead to a change in practice and in contextually interpreting their findings.

Another issue in China regarding health analytics is the quality and accessibility of data. Despite the availability of massive data sources like EHRs, wireless sensors, and medical images, the aggregation of data to produce resources that facilitate clinical research is very limited. An ideal research database is the Medical Information Mart for Intensive Care (MIMIC), a well-curated open-access database developed and maintained by the Laboratory of Computational Physiology (LCP) at the Massachusetts Institute of Technology (MIT), and supported by a vibrant research community [16]. Databases drawn from EHRs in China are smaller and accessible only to investigators internally within a hospital or organization. One notable exception is the National Scientific Data Sharing Platform for Population and Health, which encompasses or connects to various databases that include biological data, clinical data, public health data, Chinese traditional medicine data, pharmacy data, and national population and reproductive health science data [17]. These sources are analogous to US sources such as the National Inpatient Sample, which do not provide such high-resolution data as that contained in MIMIC. The following are 2 central factors seen in all countries, including China, that impede the large-scale building and dissemination of healthcare databases: (1) the previously mentioned issue of the lack of interoperability, and (2) outdated government regulations and general attitudes about data sharing and management.

As one of the largest premier hospitals in the country, and the center at which the Military No.1 project was developed, the PLAGH can be used as a reference for the state of the art in China. Each of its departments has collected vast amounts of data and can access central patient EHRs. However, there is no unified database or any direct link between the individual departments’ specialized information systems, which were designed by different vendors. Although the hospital implements several health information system standards, the individual software vendors do not consistently do so, choosing instead to focus on building software features for the various specialties. During purchase of information systems (eg, pharmacy, laboratory, clinical departments), hospital-wide integration has not been a consideration, as the recognition of EHRs as valuable research resource is only recent. As of October 2016, most of the PLAGH’s departments are working to build databases or extend and port their existing ones. A key issue that has hindered the hospital from creating a unified health information system is the undersupply of healthcare IT professionals. The hospital is only recently expanding its dedicated hospital-wide IT department. This is an even bigger issue for smaller hospitals in the country that do not have the budget for an IT team.

## The Clinical Data Conference and Health Datathon

Given these challenges, and the opportunities for growth in the utilization of EHRs for clinical research, we organized the first PLAGH-MIT clinical data conference and health datathon in Beijing, China on October 21-22, 2016.

The word datathon originates from hackathon, a short but high-energy event in which teams generate innovative, technological solutions to real-world problems. Deviating from hackathons, datathons bring together people from diverse backgrounds around data. In the context of health datathons, participants may include clinicians, data scientists, statisticians, and even patients [18].

We believe that gathering data scientists and clinicians and giving them the opportunity to explore an EHR-derived clinical database could demonstrate the value of such resources for knowledge creation and validation. As with the hackathon, the key objective of the datathon is to convince the stakeholders, who are typically holed up in their own silos, that they can accomplish so much more if they take advantage of each other’s expertise.

The MIT team has organized dozens of health hackathons and datathons around the world (Table 1) [19-21]. For outcomes, the group has been focusing on measuring affective learning and teamwork skills gained by the participants instead of metrics such as publications produced and patents for and start-up companies. The ability to work across disciplines is considered an instrumental attribute in the design, implementation, and evaluation of technological solutions to address problems in healthcare.

**Table 1:** Healthcare hackathons and datathons hosted by the Massachusetts Institute of Technology Lab for Computational Physiology.

Date	Event	Location
July 2017	Health Datathon	Singapore
June 2017	Mobile Health Hackathon	Mexico City, Mexico
May 2017	Health Datathon	Sao Paulo, Brazil
April 2017	Hacking Discrimination Hackathon	MIT^a^, United States
March 2017	Health Datathon	Melbourne, Australia
January 2017	Mobile Health Hackathon	Khon Kaen, Thailand
December 2016	Health Datathon	London, United Kingdom
October 2016	Health Datathon	Beijing, China
September 2016	Internet of Things Hackathon	Taipei, Taiwan
August 2016	Hacking Mobile Health Hackathons	MIT, United States
January 2016	Mobile Health Hackathon	Mexico City, Mexico
October 2015	Mobile Health Hackathon	Thessaloniki, Greece
September 2015	Health Datathon	MIT, United States and London, United Kingdom
July 2015	Mobile Health Hackathon	Kampala, Uganda
June 2015	Mobile Health Hackathon	Popayan, Colombia
September 2014	Health Datathon	MIT, United States, London, United Kingdom, and Paris, France
January 2014	Health Datathon	MIT, United States

^a^MIT: Massachusetts Institute of Technology.

Another notable initiative that addresses cross-disciplinary healthcare research in China is the Joint Institute for Translational and Clinical Research. Established between the University of Michigan Health System and the Peking University Health Science Center in 2010, this partnership aims to leverage the diverse expertise of researchers across both countries and universities. Members have established multiple clinician-led clinical data projects and programs such as the Biorepository and Bioinformatics Core that supports the acquisition, storage, and management of clinical information and bio-specimens [22].

Unsurprisingly, we received strong interest from many sectors as soon as the event was announced, with more than a thousand conference attendance requests on the first day of registration. Priority was given to clinicians who had previously collaborated with PLAGH. The final composition of the conference attendees was approximately one-third doctors, one-third data scientists, and one-third biomedical engineers. There were also several clinical directors, from both private and public hospitals across China. From the PLAGH, the directors of the emergency department, intensive care unit (ICU), and respiratory medicine department attended.

Disproportionately more data scientists registered for the datathon than clinicians did. This was expected and has been observed by the MIT team in events they organized across the globe. Clinicians are generally busy with patient care responsibilities and schedules that are not easy to rearrange. There is also no compelling incentive for healthcare providers to participate in research. To promote this event within the PLAGH and to recruit clinicians for the datathon, the organizers visited several departments including the emergency department, and the respiratory, cardiac and surgical ICUs. The biggest recruitment challenge within the hospital was that clinicians were not clear about their role in the datathon and had difficulty envisioning how to interface with data scientists with expertise in machine learning and signal processing. In addition, clinicians were uncertain about the types of research projects suitable for the datathon given their unfamiliarity with MIMIC-III.

The datathon centered around MIMIC-III, a de-identified database containing health related information associated with over 40,000 patients admitted into the ICUs of the Beth Israel Deaconess Medical Center between 2001 and 2012 [16]. In addition to administrative data such as transfers, discharges, and billing information, MIMIC-III contains high resolution medical data such as hourly physiological measurements, diagnoses, laboratory test results, death data collected from both the hospital and the government, and even clinical notes. A dedicated SQL server was created to allow participants to query the MIMIC-III database through a secure private network configured in the hospital. To ensure proper care was taken with the data, all participants were made to complete a training program in human research participant protection and Health Insurance Portability and Accountability Act (HIPAA) regulations beforehand. Those who are granted access agree to use it solely for population-based scientific research. They may not share it or search for specific individuals in the database.

Like prior health datathons hosted in other countries by the MIT team, the Beijing event consisted of 2 parts: a half-day conference on health data, and a full day of hands-on exploration and analysis of the clinical database.

The conference commenced with a welcome address from the PLAGH vice president Kunlun He. Dr Leo Anthony Celi discussed the opportunities from and challenges in the secondary analysis of EHRs and data sharing, based on his experiences at MIT as clinical research director of the LCP. Dr Kee Yuan Ngiam described how the National University of Singapore hospital has put health data into real-world use to establish a clinical decision support system. Dr Zhengbo Zhang described the use of numerical models applied to physiological data captured through wearables, in creating personalized diagnoses and treatments. Lastly, Dr Tom J Pollard and Dr Alistair Johnson described the evolution of the MIMIC-III database and shared their experiences in data analysis using MIMIC-III.

Research projects were proposed by PLAGH clinicians prior to the event and reviewed by both PLAGH and MIT teams in order to assess their suitability. The final approved projects were (1) total fluid balance and mortality in elderly critically ill patients; (2) serum N-terminal pro b-type natriuretic peptide (NT-proBNP) level and the duration of mechanical ventilation; (3) trends in the use of continuous renal replacement therapy in critically ill patients from 2001 through 2012; (4) variations in the treatment of hypotension according to time of day and/or day of the week; (5) the effect of age and clinical circumstances on the outcome of red blood cell transfusion in critically ill patients; and (6) the use of intra-aortic balloon pump and lactate clearance.

After the participants were split into 6 teams and assigned research questions, each team spent a full day to understand the context of the project assigned to them with input from their clinicians, extract the data from MIMIC-III, and develop data models to address their research question. Each team presented their study design and preliminary findings and shared their thoughts and experiences at the conclusion of the event. An expert panel that consisted of both computer scientists and clinicians judged the presentations and selected the winning team.

## Results of the Datathon

The majority of the datathon participants had little or no experience performing research with EHR data. Key concepts were elucidated, including the variability of the data quality due to documentation methods, how database variables are stored and distributed across multiple tables to optimize data storage, and the lack of a graphical user interface (GUI) for viewing the time series nature of majority of the variables.

Data scientists and statisticians relied on their team’s clinicians to understand the context of their assigned questions and interpret their analyses, and clinicians comfortably relied on the data scientists to perform the data extraction and analysis. Though it was the job of the data scientists to extract data, many of them had never worked with a high-resolution database, and hence extensively deferred to their team leaders from MIT. Data scientists were also aided by a collaborative code repository maintained by the LCP and hosted on GitHub. Once the data had been extracted and preprocessed, the teams were able to quickly progress through data modeling. As expected, the language barrier between some teams and the MIT team slowed down their investigations.

In the end, each of the 6 teams was able to extract their selected variables, including the outcome of interest, and perform a preliminary analysis. The expert panel gave feedback regarding each team’s choice of covariates and data models and suggested follow-up methodologies to further their clinical projects. The winning team, which investigated red blood cell transfusion, presented their coding framework that allowed them to visualize quickly the effects of adding and removing variables in the data model. This framework was well received as a useful tool with which clinicians can explore the database. Visualization software has proven helpful for non-coding clinical researchers to explore databases [23].

Participants praised the datathon’s model of providing practical experience in the design and implementation of research projects using electronic healthcare data, as opposed to previous events they had attended which only included presentations. The participants also stated that they enjoyed working with a diverse group of clinicians, data scientists, and biomedical engineers, and those from the PLAGH believed that this event would help foster interdepartmental collaboration.

## Lessons Learned Towards a Data Driven Learning Healthcare System in China

Compared with previous datathons hosted by the MIT team in the United States, United Kingdom, and France, participants were less independent in carrying out their tasks, more reliant on the event organizers, and less familiar with the concept of sharing code and data [18]. Some attendees also left skeptical about the feasibility of performing research on EHR data given the state of the art in China. The experience of and the feedback from the datathon attendees, who represented some of the leading institutions of the country, suggest that the analysis of healthcare data in China is still in its infancy.

In the end, the event was still successful in that participants were shown the promise of EHR derived healthcare databases and the potential of using them to answer clinical questions, with every group producing preliminary findings and several groups choosing to continue their projects after the event. Projects originating from past datathons have led to publications and valuable code contributions to the MIMIC code repository, a shared open access storehouse of codes for querying and analyzing MIMIC [24]. Attendees also experienced firsthand the value of cross-disciplinary collaboration, an important take-home message that rings especially true in the convoluted field of health data analytics [2,18]. The clinicians expressed enthusiasm about the possibility of holding future datathons with databases constructed from the PLAGH. In conjunction with promoting the value of healthcare databases, the event also served its purpose of highlighting the challenges and limitations of building and learning from them, and the need to invest heavily in the development of such resources. Following the event, the PLAGH launched a series of hospital-wide data merging and data warehouse construction projects. In particular, it is building an ICU database with the intention of publishing it and using it for the next datathon. In addition, the biomedical engineering department is preparing its new course: Secondary Analysis of EHRs for the hospital’s graduate school. This course teaches skills in data extraction, processing, and modeling, training new researchers to leverage the value of big data in healthcare.

As the value of health data analytics becomes more apparent, it is likely that Chinese institutions will dedicate more funding and workers into building EHR systems and creating databases from them [25]. The tier 3 and the tier 2 and below hospitals that participated in the 2015 CHIMA survey plan to spend an average of RMB $15 million and RMB $4.4 million (US $2 million and $0.6 million, respectively) on their digital information systems in 2016 and 2017. In addition, 96% of them have already established a department dedicated to the digitization of their health records [13].

However, the speed and efficiency at which healthcare databases are built, and the volume and quality research that ultimately arises from them, will largely depend on institutions’ understanding of health data—what it represents, its structure, the way it is captured, and the inherent biases as a result. The goals of digital health information systems are to improve the efficiency of operations (88%), to reduce clinical errors (85%), to reduce operating costs (67%), and to improve patient satisfaction (60%) [13]. Notably excluded from the list is the use of data by health organizations to learn continuously from the way care is delivered to maximize patient outcomes.

The MIT LCP and the PLAGH will continue to organize events to demonstrate the benefits of EHR databases to Chinese stakeholders: the government, industry, hospitals and clinics, but most of all, the patients. Only when these stakeholders appreciate their value, will there be a push to invest in building such resources. In addition, these events promote the need to train more technical specialists to build and manage healthcare databases and the clinicians and data scientists who understand how to use them.

Last, we cannot emphasize enough the importance of collaboration and data sharing across healthcare organizations. The lack of data standards and interoperability between EHRs has greatly impeded learning from routinely collected health data [26,27]. This, along with the culture of researchers hoarding data and working in silos, results in waste and inefficiencies in biomedical research. Hence members from government, industry, and academia must coordinate their efforts to develop and implement common data standards and adopt policies that promote data sharing.

The late emergence of EHRs in China relative to the United States provides China a unique opportunity to learn from past experiences, including failures in developing an efficient digital healthcare infrastructure. Obstacles and challenges, if addressed and circumvented, could facilitate country-wide EHR analysis and reduce institutional hoarding of data, clinicians and data scientists continuing to work in silos, and the lack of incentives for data systems interoperability. Experts should continue to push for a culture of data sharing and collaboration and expound the vast potential and practical limitations of the secondary analysis of EHRs to make data-driven learning in healthcare a reality in China.

## Abbreviations

CHIMA: China Hospital Information Management Association
EHR: electronic health record
ICU: intensive care unit
IT: information technology
LCP: Lab for Computational Physiology
MIT: Massachusetts Institute of Technology
MIMIC-III: Medical Information Mart for Intensive Care
NHFPC: National Health and Family Planning Commission of the People’s Republic of China
PLAGH: People’s Liberation Army General Hospital
RCT: randomized control trial
